# Effect of Quercetin on ABCC6 Transporter: Implication in HepG2 Migration

**DOI:** 10.3390/ijms22083871

**Published:** 2021-04-08

**Authors:** Vittorio Abruzzese, Ilenia Matera, Fabio Martinelli, Monica Carmosino, Prashant Koshal, Luigi Milella, Faustino Bisaccia, Angela Ostuni

**Affiliations:** Department of Sciences, University of Basilicata, 85100 Potenza, Italy; v.abruzz@hotmail.it (V.A.); ilenia.matera@gmail.com (I.M.); fabiomartinelli@alice.it (F.M.); monica.carmosino@unibas.it (M.C.); prashantkoshal240@gmail.com (P.K.); luigi.milella@unibas.it (L.M.)

**Keywords:** quercetin, ABCC6, HepG2, cytoskeleton, cell migration, PI3K/AKT signaling

## Abstract

Quercetin is a member of the flavonoid group of compounds, which is abundantly present in various dietary sources. It has excellent antioxidant properties and anti-inflammatory activity and is very effective as an anti-cancer agent against various types of tumors, both in vivo and in vitro. Quercetin has been also reported to modulate the activity of some members of the multidrug-resistance transporters family, such as P-gp, ABCC1, ABCC2, and ABCG2, and the activity of ecto-5′-nucleotidase (NT5E/CD73), a key regulator in some tumor processes such as invasion, migration, and metastasis. In this study, we investigated the effect of Quercetin on ABCC6 expression in HepG2 cells. ABCC6 is a member of the superfamily of ATP-binding cassette (ABC) transporters, poorly involved in drug resistance, whose mutations cause pseudoxanthoma elasticum, an inherited disease characterized by ectopic calcification of soft connective tissues. Recently, it has been reported that ABCC6 contributes to cytoskeleton rearrangements and HepG2 cell motility through purinergic signaling. Gene and protein expression were evaluated by quantitative Reverse-Transcription PCR (RT-qPCR) and western blot, respectively. Actin cytoskeleton dynamics was evaluated by laser confocal microscopy using fluorophore-conjugated phalloidin. Cell motility was analyzed by an in vitro wound-healing migration assay. We propose that ABCC6 expression may be controlled by the AKT pathway as part of an adaptative response to oxidative stress, which can be mitigated by the use of Quercetin-like flavonoids.

## 1. Introduction

Quercetin is a member of the flavonoid group of compounds, which is abundantly present in various dietary sources like vegetables, fruits, spices, tea, and wine. Quercetin is usually present as a glycoside, though the aglycone moiety is absorbed after deglycosylation in the gut. Its daily intake has been estimated to be about 6–18 mg in the United States, China, and Netherlands. Its bioavailability is considered low and varies from 2% to 44% in different studies, due to its poor solubility in water and to intestinal and biliary excretion, which limit its absorption [1].

Thanks to its ability to bind transition metal ions [2] and to scavenge free radicals, Quercetin has excellent antioxidant properties attributed to the catechol group in the B ring and the OH group at position 3 of the A ring [3]. As a radical scavenger, Quercetin is effective against O_2_^−^ and ONOO^−^ and can terminate lipid peroxidation, which has deleterious effects especially on the cardiovascular and nervous systems [4,5]. Quercetin also shows good anti-inflammatory activity by reducing Lipopolysaccharide (LPS)-induced expression of Tumor Necrosis Factor (TNF) TNF-α and Interleukin (IL)-1α [6] and by preventing the production of the inflammatory enzymes lipoxygenases (LOX) and cyclooxygenases (COX) [7].

Moreover, Quercetin has been found to be useful in cancer prevention, and very effective as an anti-cancer agent against various types of tumors, both in vivo and in vitro, such as breast, colon, liver, pancreas, lung, prostate, bladder, bone, and blood cancers [8]. Multiple mechanisms and pathways are involved, from cell cycle arrest to induction of apoptosis and autophagy [9]. The progression and metastatic potential of solid tumors are also affected through the inhibition of angiogenesis in a Vascular endothelial growth factor receptor 2 (VEGFR 2)-dependent manner [10,11] and by targeting Epithelial-to-Mesenchymal transition (EMT) [12,13] and Metalloproteinases (MMP)-mediated remodeling of the Extracellular Matrix (ECM) [14,15]. Furthermore, Quercetin has been reported to inhibit the activity of ecto-5′-nucleotidase (NT5E) [16,17], a key enzyme in the purinergic signaling pathway [18] and in the remodeling of ECM components in cancer invasion [19]. It has been known for a long time that flavonoids can modulate both expression and activity of many ABC transporters, with some relevant impact on drug resistance [20,21,22]. Quercetin has been also reported to modulate the activity of some members of the multidrug-resistance transporters family such as P-gp [23,24,25,26,27,28], ABCC1 [27,29], ABCC2, and ABCG2 [27]. ABCC6 is a member of the C subfamily of ATP-binding cassette transporters mainly expressed in liver and kidney, poorly involved in drug resistance [30,31,32]. We recently demonstrated that ABCC6 silencing or its inhibition by the uricosuric drug probenecid, through the modulation of the purinergic system, leads to cytoskeleton rearrangement and reduced motility of HepG2 cells, thus identifying it as a potential target for anti-metastatic treatment [33]. The aim of this study was to study the effect of Quercetin on ABCC6 expression. Quercetin decreased the expression of ABCC6 through the regulation of the AKT signaling pathway, thus also contributing to cytoskeleton rearrangement and reduced cells motility.

## 2. Results

### 2.1. Effect of Quercetin on Cells Viability and Reactive Oxygen Species Accumulation

**Figure 1:**
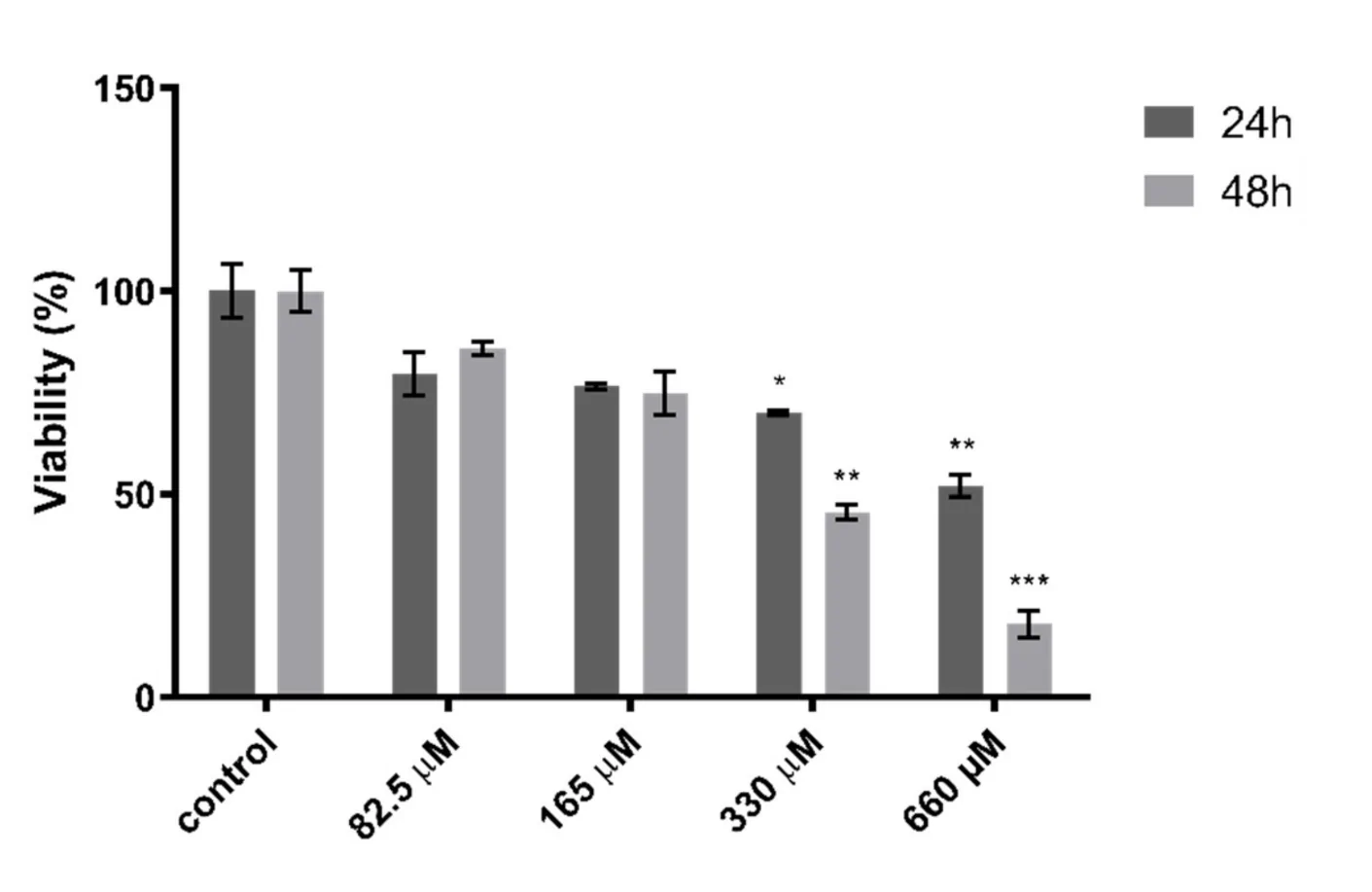
Effect of quercetin on HepG2 cells viability. Cells were treated with Quercetin at concentrations of 82.5, 165, 330, and 660 μM for 24 and 48 h. Data are expressed as a percentage of the control group ± the standard error of the mean of three replicates from three independent experiments. Statistical significance was assessed by multiple *t* test followed by Holm–Sidak correction for multiple comparisons; * *p* < 0.05, ** *p* < 0.01, *** *p* < 0.001.

In order to assess the effect of Quercetin on cells viability, an MTT assay was performed on HepG2 cells treated with different concentrations of Quercetin, ranging from 660 to 82.5 µM, for 24 and 48 h. We found that Quercetin affected cell viability in a concentration- and time-dependent manner. However, a concentration of 165 μM was chosen for further experiments, as it did not cause significant cell toxicity at both 24 and 48 h of treatment (Figure 1).

**Figure 2:**
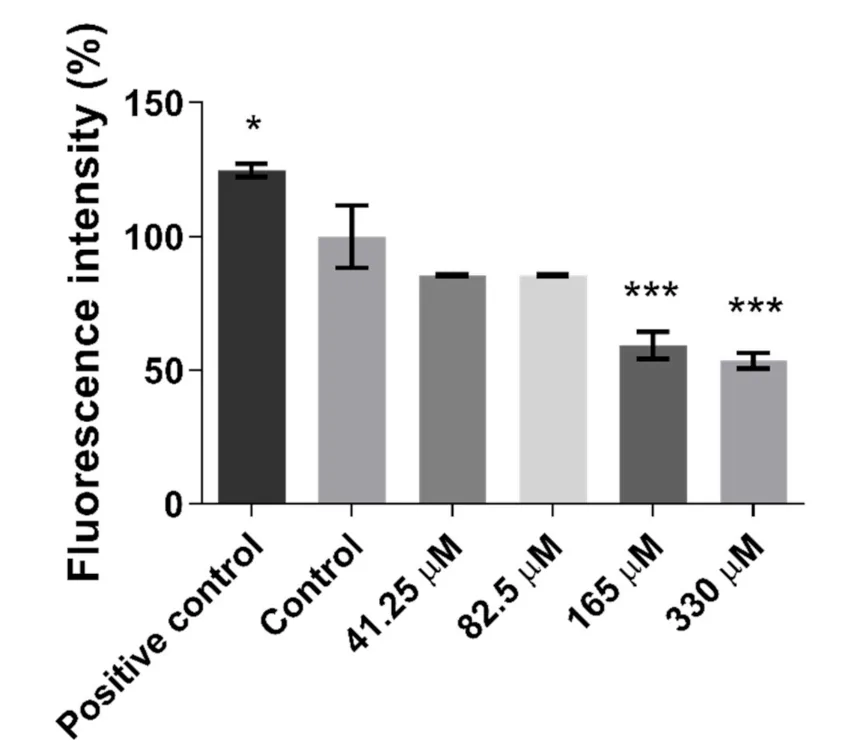
Effect of Quercetin on the intracellular level of Reactive Oxygen Species (ROS) in HepG2 cells. Cells were treated with Quercetin at concentration of 41.25, 82.5, 165, and 330 mM for 24 h. A two-hour pre-incubation with 500 μM H_2_O_2_ was used as a positive control. Data are expressed as a percentage of the control group ± the standard error of the mean of three replicates of three independent experiments. Comparisons between treatments and control groups were performed by one-way ANOVA followed by Dunnett post-hoc correction; * *p* < 0.05; *** *p* < 0.001.

In order to assess the antioxidant activity of Quercetin on HepG2 cells, a 2′,7′-Dichlorofluorescein (DCF) assay was performed to evaluate its effect on intracellular Reactive Oxygen Species (ROS) accumulation. As shown in Figure 2, Quercetin at a concentration of 165 μM significantly lowered the oxidative stress, with a reduction of intracellular ROS level greater than 40% as compared to the control cells.

### 2.2. Effect of Quercetin on Gene Expression

**Figure 3:**
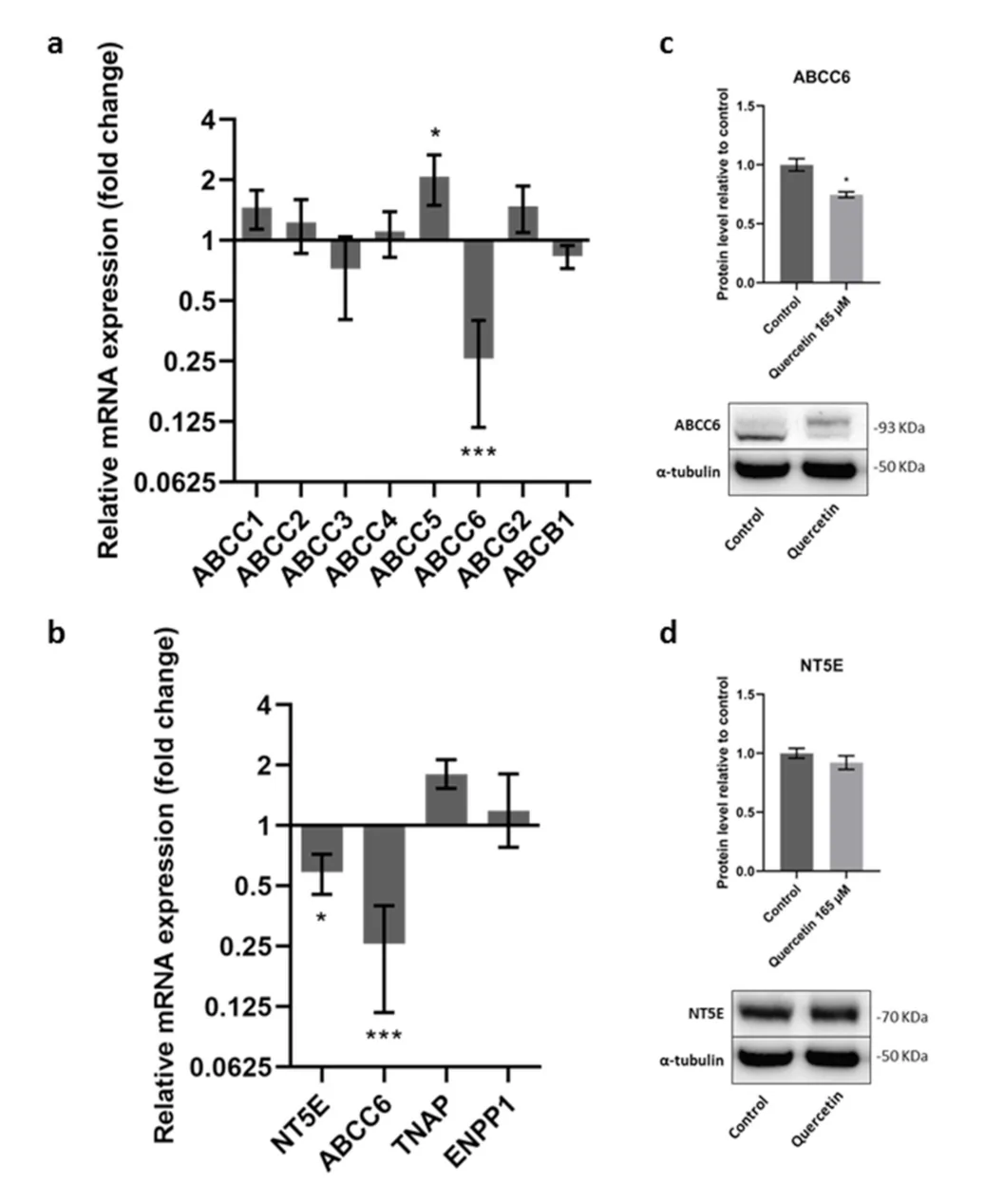
Effect of Quercetin on mRNA expression of some relevant ABC transporters (**a**) and on genes involved in the purinergic pathway (**b**). HepG2 cells were treated with Quercetin (165 μM) for 24 h. Cells treated with 0.25% DMSO were used as a control. Results are expressed as the mean and 95% confidence interval of three different experiments. Statistical analysis was performed on ΔCt values by using multiple T-test followed by Holm–Sidak correction for multiple comparisons; * *p* < 0.05; *** *p* < 0.001. Effect of Quercetin on ABCC6 (**c**) and NT5E (**d**) protein expression. Results are expressed as the mean ± the standard error of three independent experiments. Results were analyzed by Student’s *t* test; * *p* < 0.05.

In order to verify whether Quercetin was able to affect the expression of some relevant ABC transporters in HepG2 cells, an RT-PCR experiment was carried out. As shown in Figure 3a, no significant variation was found in such gene expression, with the only exception of ABCC6 and ABCC5. Fold change in mRNA expression was, respectively, 0.26 (95% C.I.: 0.40; 0.12) and 2.08 (95% C.I.: 2.66; 1.49). Previously, we demonstrated that NT5E expression is regulated by ABCC6, which in turn supplies ATP to feed the purinergic system [34,35]. In panel b of Figure 3, the effect of Quercetin on genes involved in the purinergic system is shown. An increase in tissue-nonspecific alkaline phosphatase (TNAP) was found (fold change 1.81; 95% C.I.: 2.13; 1.53), while NT5E expression was significantly decreased (fold change 0.59; 95% C.I.: 0.72; 0.45). Western blot analysis showed that Quercetin decreased ABCC6 protein levels but not the expression of NT5E (Figure 3c,d).

### 2.3. Quercetin Induces a Rearrangement of the Actin Cytoskeleton

**Figure 4:**
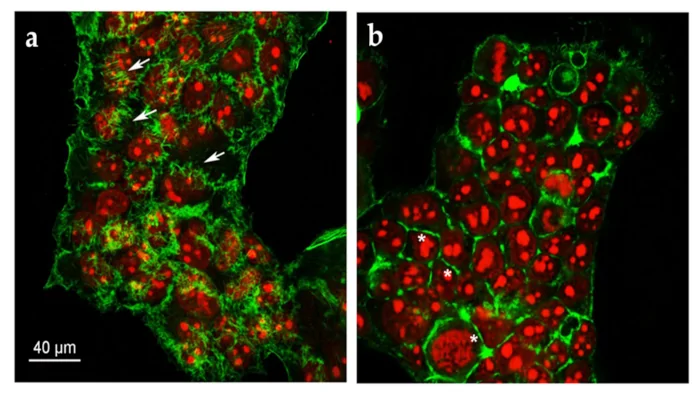
Representative confocal image of HepG2 cells treated with (**a**) 0.25% DMSO; (**b**) 165 µM Quercetin for 24 h. F-actin and nuclei were stained with Phalloidin Alexa Fluor 488 and propidium iodide, respectively.

Cell motility is a key factor in a variety of pathophysiological processes, such as tumor invasion and metastasis. Cell movement is widely considered a very complex phenomenon driven by a finely coordinate rearrangement of actin filaments in the cytoskeleton. This is a highly dynamic process in which protrusive structures form at the leading hedge of motile cells, while at the opposite extremity, the body of the cells is retracting and loosening adhesion to adjacent cells and to the matrix scaffold. These typical structures, named lamellipodia and filopodia, are generated by the polymerization of filamentous actin and further organization in tight bundles (filopodia) or cross-woven webs (lamellipodia), in which filaments are oriented at a certain angle to the direction of movement [36,37]. In order to evaluate if any changes occurred in the organization of the cytoskeleton following the treatment with Quercetin, immunofluorescence experiments coupled to laser confocal microscopy were carried out. HepG2 cells were grown on a coverslip and stained with a derivate of phalloidin, which binds in a specific manner to the actin filaments, conjugated with a fluorescent dye. Many filopodia were observed in HepG2 control cells (Figure 4a, arrows), while these structures were almost completely absent in Quercetin-treated cells (Figure 4b, stars), suggesting an inhibition of cell motility in Quercetin-treated cells.

### 2.4. Quercetin Affects HepG2 Cells’ Migration Rate

**Figure 5:**
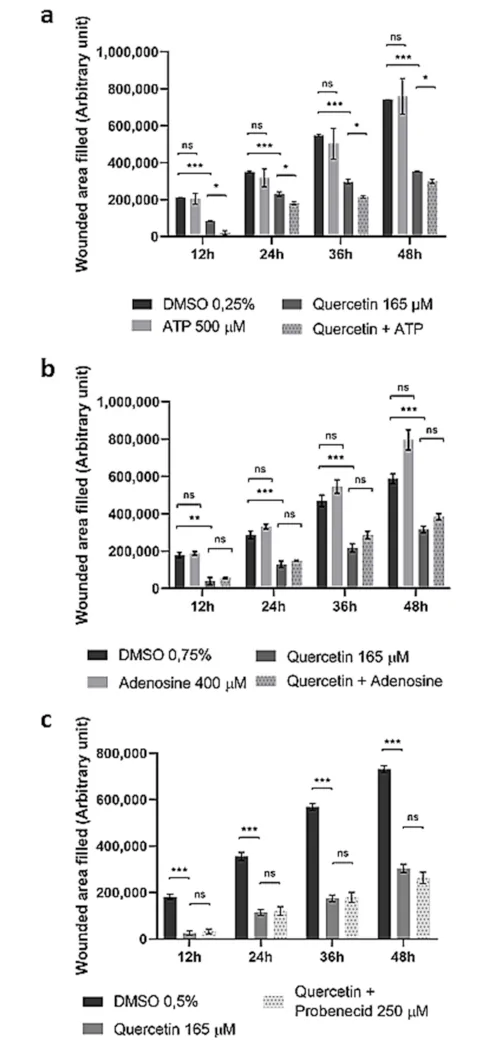
Effect of Quercetin on HepG2 cells’ migration rate. Cells were treated with Quercetin (165 μM) for 12 h. Then, a scratch was made in the cell monolayer, and pictures were taken every 12 h, while the cells were still kept in contact with Quercetin. DMSO-treated cells were used as a control. ATP 500 μM (**a**), Adenosine 400 μM (**b**), or Probenecid 250 μM (**c**) was added to both control and Quercetin-treated cells. Data are expressed as the mean and standard error of three different experiments. Statistical significance was assessed by multiple *t* test followed by Holm–Sidak correction for multiple comparisons; * *p* < 0.05; ** *p* < 0.01; *** *p* < 0.001. Representative pictures of the scratches taken at different times are shown in Supplementary Figure S1.

Cytoskeletal rearrangement is closely related to cell migration. To study the effect of Quercetin on cell migration in HepG2 cells, an in vitro scratch test was performed. The scratch test is an easy, fast, accurate, and highly reproducible method to assess cell collective migration [38]. As shown in Figure 5, Quercetin significantly reduced the migration rate in HepG2 cells. Unlike what was previously observed in Probenecid-treated or ABCC6-silenced cells, neither ATP (Figure 5a) nor Adenosine (Figure 5b) restored cells motility. In addition, when ATP was used in combination with Quercetin, a further decrease in migration was observed. No effect on cell migration was detected when Probenecid was used in combination with Quercetin (Figure 5c).

### 2.5. Effect of Quercetin on MAPK/ERK and PI3K/AKT Pathways

**Figure 6:**
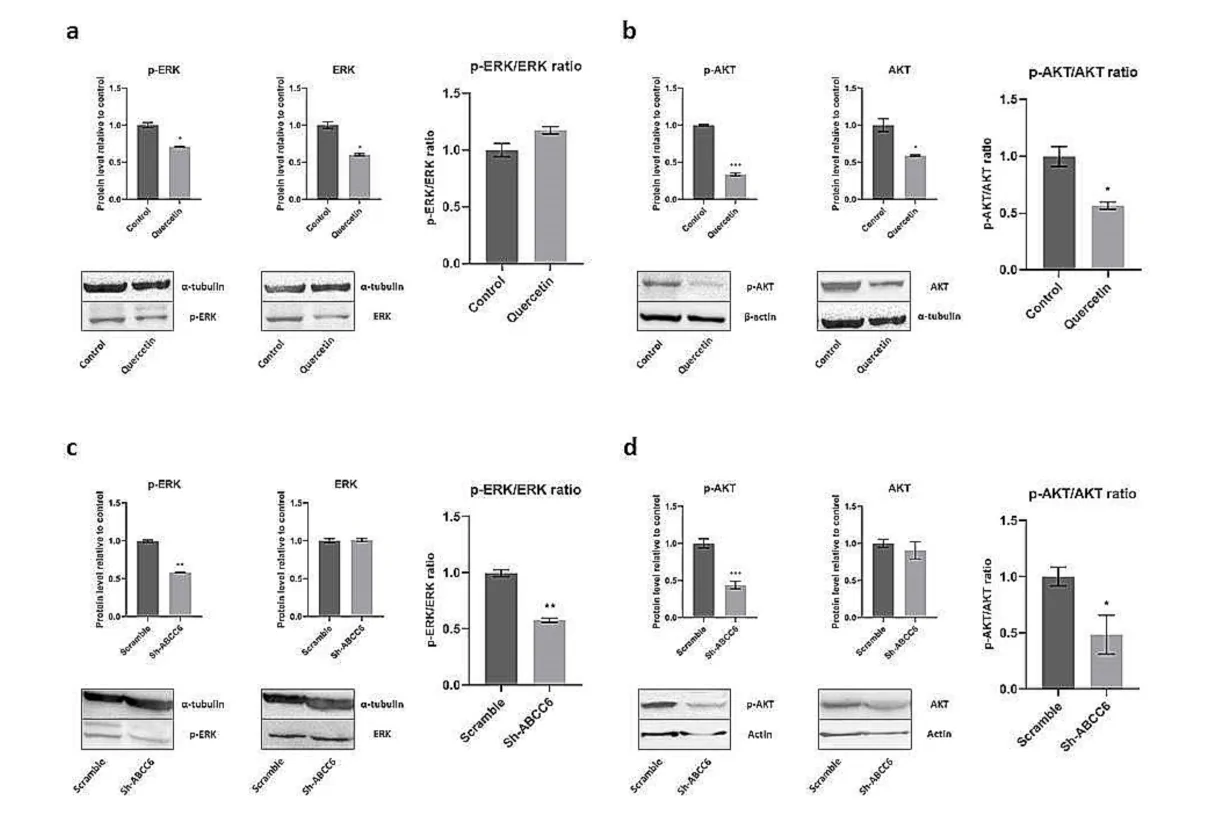
Effect of Quercetin treatment (**a**,**b**) or ABCC6 silencing (**c**,**d**) on phosphorylated AKT and ERK. The ratios between phosphorylated and total ERK (**a**,**c**) and between phosphorylated and total AKT (**b**,**d**) were determined by comparing the intensities of the immunoreactive bands obtained by using specific antibodies. Cells treated with DMSO 0.25% or scrambled sh-RNA were used as controls for HepG2 cells treated with Quercetin and subjected to ABCC6 knockdown, respectively. α-tubulin or β-actin were used as a loading control. Data are presented as the mean and the standard error of the mean of three independent experiments. Results were analyzed by Student’s *t* test; * *p* < 0.05; ** *p* < 0.01; *** *p* < 0.001.

In order to shed light on the molecular mechanism involved in Quercetin-mediated impairing of HepG2 cell motility, we investigated two major signaling pathways, known to be among the main regulators of cell motility, namely, phosphoinositide 3 kinase (PI3K)/AKT (also known as Protein Kinase B, PKB) and extracellular signal-regulated kinase (ERK) pathways [39,40]. In Quercetin-treated cells, no changes in p-ERK/ERK ratio were detected (Figure 6a); on the contrary, the level of p-AKT was reduced (Figure 6b). Interestingly, in ABCC6-silenced (Sh-ABCC6) cells, a decrease of both phosphorylated kinases was observed (Figure 6c,d).

## 3. Discussion

Flavonoids modulate both the activity and the expression of several ABC transporters [20,21,22]. Most studies have been focused on P-gp, BCRP, and MRP1, which have a major role in drug resistance, but very limited information is available on different transporters and, specifically, on ABCC6, to our knowledge [41].

Quercetin is one of the most abundant flavonoids derived from plants, whose antitumor activity in hepatocarcinoma was recently systematically reviewed [42]. In the present study, interestingly, among the considered ABCs, we found the most significant decrease in ABCC6 expression (Figure 3a), which is only marginally involved in drug resistance.

In previous studies, we found that pharmacological inhibition or silencing of ABCC6 in HepG2 cells could contribute to cytoskeleton rearrangement and cell motility by reducing the availability of ATP to feed the extracellular purinergic purine pool [33]. Therefore, we designed experiments to test the hypothesis that Quercetin, inhibiting ABCC6 expression, could impair cell motility by acting on the purinergic system. Although the treatment with Quercetin significantly reduced the migration rate of HepG2 cells, neither ATP nor Adenosine restored motility. Moreover, the further decrease of the migration rate when ATP was used in combination with Quercetin could be explained by the inhibition of NT5E enzymatic activity, previously assessed [16,17]: most likely, in the presence of Quercetin, AMP accumulates and exerts an additional inhibitory effect on cell motility by acting on different purine receptors. The addition of Probenecid in combination with Quercetin did not modify cell migration, probably because the effect of Quercetin includes the effect of Probenecid on the purinergic system.

All together, these results suggest that the effect of Quercetin on cell motility may be due to the involvement of other targets and pathways, as indicated by its pleiotropic activity. It is widely accepted that MAPK and AKT are among major pathways involved in the control of tumor cells’ proliferation and motility [39,40]. It is known that Quercetin suppresses the migration of some Hepato Cellular Carcinoma (HCC)-derived cells by inhibiting the signaling pathway of AKT [42]; we also confirmed this effect on HepG2 cells. Interestingly, we observed the shutdown of both pathways in ABCC6-silenced HepG2 cells, probably as a consequence of a reduction of adenosine signaling, mediated by the purinergic pathway. In conclusion, both silenced cells and cells treated with Quercetin exhibited downregulation of the AKT pathway, thus suggesting that Quercetin could cause the reduction of ABCC6 expression through the downregulation of phosphorylated AKT.

Activation of AKT plays a key role in the cell response to oxidative stress, by inducing the expression of target genes, which support tumor cells’ survival, as well as resistance to chemotherapy [43,44]. In the present study, we used a concentration of Quercetin associated with strong antioxidant activity. Therefore, we propose that ABCC6 expression may be controlled by AKT activity as part of cells’ adaptative response to oxidative stress, which can be mitigated by the use of Quercetin-like flavonoids. Indeed, the downregulation of phosphorylated AKT and the removal of oxidative stress lead to decreased expression of ABCC6 transport activity and may produce a senescence-like phenotype in cancer cells, which we previously observed in ABCC6-silenced cells [45], thus through a cell pathway independent of the purinergic system.

In any case, treatment of HepG2 cells with Quercetin showed once more a clear relationship among ABCC6 downregulation, cytoskeleton rearrangement, and motility impairment. This effect on ABCC6 expression could be part of Quercetin antitumor and anti-metastatic potential, especially in those tumors with a high expression of ABCC6. However, since the lack of ABCC6 transport activity is the cause of ectopic mineralization in Pseudoxanthoma elasticum (PXE), the potential of harm deriving from reducing its activity should be carefully evaluated. This is unlikely to happen when food is the only source of Quercetin, since the daily intake is limited, but it can represent a not-so-far-from-real eventuality when Quercetin is used as a dietary supplement, a use that is acquiring a growing popularity.

## 4. Materials and Methods

### 4.1. Cell Culture and Treatments

Human hepatoblastoma cells (HepG2) were grown in Dulbecco’s modified Eagle’s medium (DMEM) with a high glucose concentration (4.5 g/L), to which 10% fetal bovine serum (FBS), 2 mM L- glutamine, 100 U/mL penicillin, and 100 µg/mL streptomycin were added. Cells were cultured at 37 °C, in a water-saturated atmosphere with 5% CO_2_. Quercetin was dissolved in DMSO at a concentration of 20 mg/mL as a stock solution. The final concentration of DMSO in cell treatments did not exceed 0.25% *v/v*. Control cells were treated at the same final concentration of DMSO (vehicle). All compounds were purchased from Sigma (Sigma, Saint Louis, MO, USA).

### 4.2. Generation of STABLE ABCC6 Knockdown HepG2 Cells

In order to silence ABCC6 expression in HepG2 cells, the shRNA technology was used by infecting cells with lentiviral particles, (vector purchased from Cyagen Biosciences (Santa Clara, CA, USA). EGFP fluorescence was used as a control for successful infection. In 12-well plates, cells were seeded at a density of 1.5 × 10^5^/well. After 24 h, a suspension of lentiviral particles at a suitable multiplicity of infection (MOI) of 10 packed with plasmid vectors containing ABCC6-shRNA or scrambled-shRNA for negative control was added. A preliminary 12-day selection with puromycin 2 µg/mL to remove non-infected cells was followed by clone selection with cloning cylinders in 200 mm plates. Clones with at least a 75% knockdown expression were used for further analysis.

### 4.3. Viability Assay

Cell viability was assessed by the MTT (3-(4, 5-dimethyl thiazol-2yl)-2, 5-diphenyl tetrazolium bromide) assay. In this experiment, 2 × 10^4^ cells were seeded in each well of a 96-well plate. After 24 h, the cells were treated with progressive dilutions of Quercetin, ranging from 660 to 82.5 μM for 24 or 48 h, then incubated with fresh medium containing 0.75 mg/mL MTT for 4 h at 37 °C. The formazan crystals were finally dissolved for 1 h at room temperature on agitation in a mixture 1:1 of DMSO and isopropanol with 1% of Triton X-100. The viability of cells was assessed by comparing the light absorbance at 570 nm, after subtraction of the background at 630 nm, of treated and control cells (treated only with vehicle DMSO), defined as 100% cell viability. Spectrophotometric assays were performed using a microplate reader (Multiskan TM GO Microplate Spectrophotometer, Thermo Scientific, Waltham, MA, USA). Experiments were conducted in triplicate.

### 4.4. Intracellular ROS Assay

The antioxidant activity of Quercetin in HepG2 cells was assessed by the 2′,7′-dichlorofluorescein assay. In this experiment, 1 × 10^4^ cells were seeded in each well of a 96-well polystyrene black plate with clear bottom. After 24 h, the cells were treated with progressive dilutions of Quercetin, ranging from 330 to 41.25 μM for 24 h. Then, the cells were incubated with dichlorofluoresceindiacetate (DCFH-DA) at a final concentration of 10 µM in PBS for 30′ at 37 °C, and fluorescence was measured by using a GloMaxMultiDetection System (Promega, Madison, WI, USA) equipped with a blue filter (ex.:490 nm; em.: 510–570 nm). As a positive control of ROS presence, a 2 h treatment prior to DCFH-DA addition was used. Results are presented as a percentage of the negative control (cells treated with the vehicle DMSO only). Each treatment was performed in triplicate.

### 4.5. Real-Time PCR

RNA extraction was performed by using the Quick-RNA MiniPrep kit (Zymo Research, Irvine, CA, USA). RNA was then retrotranscripted to cDNA using random primers and the High-Capacity cDNA Reverse Transcription kit (Applied Biosystem, Waltham, MA, USA). Total cDNA was amplified using iTaqTM Universal SYBR Green Supermix (Bio-Rad, Waltham, MA, USA) with the 7500 Fast Real-Time PCR System (Applied Biosystems). Each primer used here was designed so to span exon–exon junctions in order to prevent any unwanted genomic DNA amplification (Table 1).

The comparative threshold cycle method (ΔΔCt) was used to quantify the relative amounts of product transcripts, with β-actin as endogenous reference control. The specificity of amplicons was confirmed by melting curve analysis. Each test was performed at least in triplicate.

### 4.6. Western Blot Analysis

HepG2 cells were suspended by pipetting in Laemmli sample buffer (60 mM Tris–HCl pH 6.8, 10% glycerol, 2% SDS, 1% β-mercaptoethanol, and 0.002% bromophenol blue) supplemented with a proteases and phosphatases inhibitors cocktail and lysed by two cycles of sonication for one minute on ice. Then lysates were resolved on 8 or 10% SDS-PAGE gels, and proteins were transferred to nitrocellulose membranes. The membranes were blocked for 1 h with saturation buffer (5% nonfat dried milk in PBS or TBS with 0.05 or 0.1% Tween 20, respectively) and then probed with primary antibodies overnight at 4 °C: 1:1000 anti-α-tubulin (T9026, Sigma-Aldrich, Darmstadt, Germany), 1:400 anti-β-actin (A5441, Sigma-Aldrich), 1:1000 anti-AKT (pan) (C67E7, Cell Signaling), 1:2000 anti-phospho-AKT (Ser 473) (D9E, Cell Signaling), 1 μg/mL anti-ERK 1/2 (710214, ThermoFisher Scientific), 1 µg/mL anti-phospho-ERK 1/2 (Thr185, Tyr187) (710214, ThermoFisher Scientific), 1:50 anti-ABCC6 (MA1-26542, ThermoFisher Scientific), 1:250 anti-CD73 (1D7, ThermoFisher Scientific). The membranes were washed three times with PBST or TBST and incubated with the appropriate horseradish peroxidase- conjugated secondary antibodies at room temperature for 1 h, and signal were visualized by the ECL™ Western Blotting Detection Reagents (GE Healthcare, Chicago, IL, USA) or the SuperSignal™ West Pico PLUS Chemiluminescent Substrate (Thermo Scientific), using the Chemidoc TM XRS detection system equipped with Image Lab Software for image acquisition (BioRad, Hercules, CA, USA). Densitometric analysis was performed by using GelAnalizer 2010 software (Debrecen, Hungary). Protein expression level in control samples was taken as 100%. Each result was expressed as a percentage of the value of the control sample. Each test was repeated three times.

### 4.7. Confocal Fluorescence Microscopy

HepG2 cells (1.5 × 10^5^) were grown on coverslips in the presence of 165 µM Quercetin for 24 h. Staining of nuclei and F-actin was performed with propidium iodide and Phalloidin Alexa Fluor 488, as previously described [33].

### 4.8. Migration Assay

Cell migration rate was evaluated by an in vitro wound-healing assay. HepG2 cells (1 × 10^6^) were seeded in a 6-well plate and cultured in DMEM containing 10% FBS to obtain a nearly confluent cell monolayer. Cells were there treated with either 165 µM Quercetin or DMSO as control in the presence or absence of 500 µM ATP or 400 µM adenosine for 12 h in DMEM containing 10% FBS. Then, a linear wound was generated in the cellular monolayer with a sterile 10 µL plastic pipette tip. Any cellular debris was removed by washing with PBS, and the medium replaced with 2 mL of DMEM with 1% FBS still containing 165 µM Quercetin or 0.25% DMSO in the presence or absence of ATP or adenosine. The cells were incubated at 37 °C, and pictures of the scratch were taken every 12 h by using a Nikon Eclipse TS 100 inverted microscope equipped with a Nikon Coolpix P6000 digital camera (objective magnification 10×) (Minato, Tokyo, Japan). The scratch area was measured by the free processing software Icy image.

### 4.9. Statistical Analysis

All assays were performed at least three times, independently. The standard error of the mean or the 95% confidence interval was reported as a measure of variability. Statistical analysis was performed by Student’s *t* test or ANOVA, using GraphPad Prism software. An alpha level of 0.05 was chosen to define statistical significance.

## Figures and Tables

**Table 1:** List of primers used in this study.

Gene	Primer Forward	Primer Reverse
Β-actin	5′-CCTGGCACCCAGCACAAT-3′	5′-GCCGATCCACACGGAGTACT-3′
ABCC1	5′-GCTGATGGAGGCTGACAAGG-3′	5′-GATGCTGAGGAAGGAGATGAAGAG-3′
ABCC2	5′-CCCTTGTCCTGGAAGATGTT-3′	5′-AGAGCCTTCATCAACCAGG-3′
ABCC3	5′-CCACACCACAACCACCTTCAC-3′	5′-CTCGGCGTCCAGCACATTG-3′
ABCC4	5′-GCACACCAGGATTTACATTCAGAG-3′	5-CCAGACGGACGGCAAACC-3′
ABCC5	5′-CCACCATCCACGCCTACAATAAAG-3′,	5′-ACAGCCAGCCACCGCATC-3′
ABCC6	5′-ATCACTGATCCTTCCATCTTG-3′	5′-ACCAGCGACACAGAGAAGAGG-3′
ABCG2	5′-ATCACTGATCCTTCCATCTTG-3′	5′-GCTTAGACATCCTTTTCAGG-3′
ABCB1	5′-CCTTCAGGGTTTCACATTTGG-3′	5′-ACTCACATCCTGTCTGAGCA-3′
NT5E	5′-GGGCGGAAGGTTCCTGTAG-3′	5′GAGGAGCCATCCAGATAGACA-3′
TNAP	5′-TTTCCACTCTGCGCCGCTACC-3′	5′-CGCCTGTAATCCCAGCACTTT-5′
ENPP1	5′-CGCCTGTAATCCCAGCACTTT-3′	5′-ATGGACAGGACTAAGAGGAATTCTAAA-5′

## Supplementary Materials

The following are available online at https://www.mdpi.com/article/10.3390/ijms22083871/s1, Figure S1: Representative pictures of the scratches taken at different times.
