# Supplementary material for: Effect of Quercetin on ABCC6 Transporter: Implication in HepG2 Migration
Source: Int J Mol Sci. 2021 Apr 8;22(8):3871. doi: 10.3390/ijms22083871 (PMC8069417; doi:10.3390/ijms22083871)
Supplement: Supplementary file 1 [file ijms-22-03871-s001.pdf]

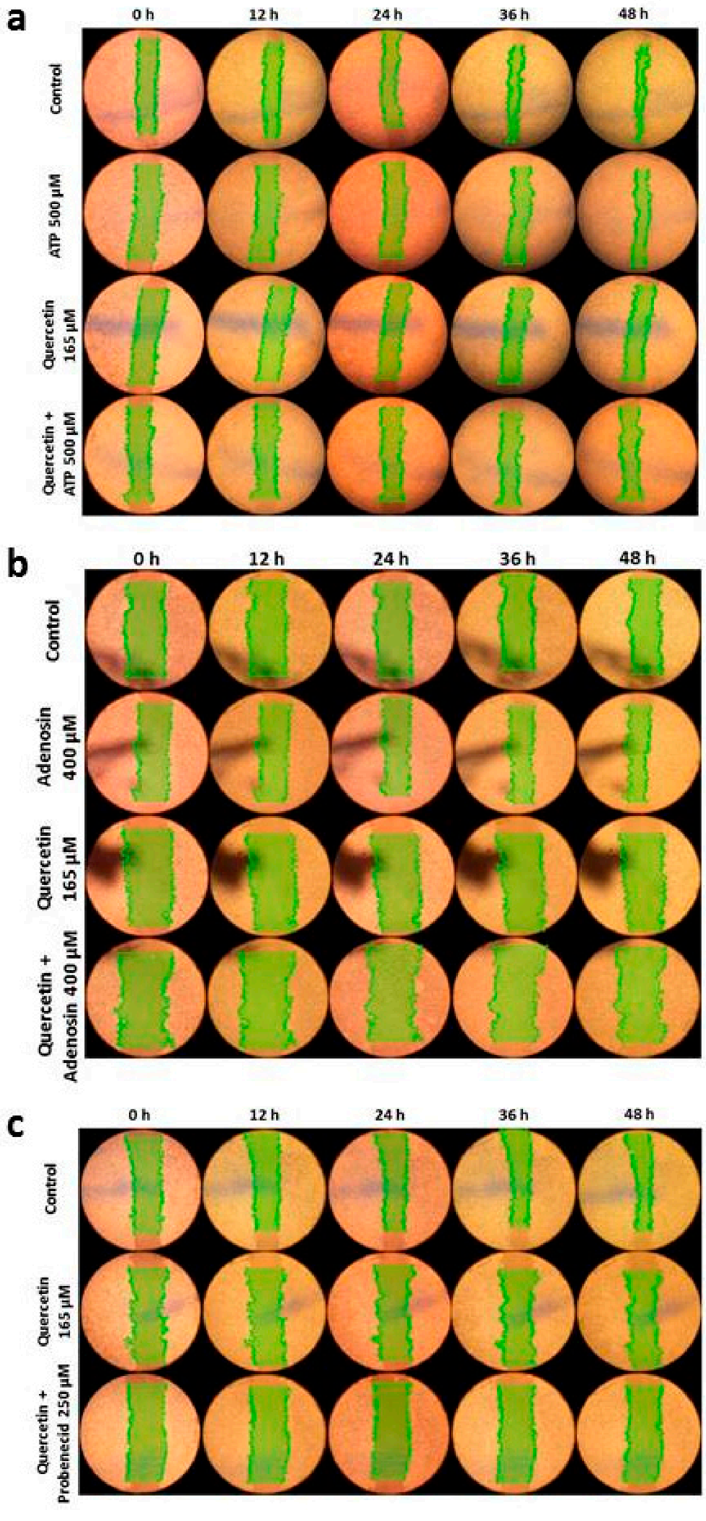

Supplementary figure S1. Representative pictures of the scratch assay. Cells were treated with test substances for 12h in culture medium containing 10% FBS. Then, a linear scratch was made in the cell monolayer with a plastic pipette tip and medium with 1% FBS and test molecules was added. Pictures were taken immediately (T0) and every 12h for two days.
